# Flavonoid and Antioxidant Capacity of Propolis Prediction Using Near Infrared Spectroscopy

**DOI:** 10.3390/s17071647

**Published:** 2017-07-18

**Authors:** Eddy Betances-Salcedo, Isabel Revilla, Ana M. Vivar-Quintana, M. Inmaculada González-Martín

**Affiliations:** 1 Department of Analytical and Food Chemistry, Faculty of Chemistry, University of Salamanca, Plaza de la Merced, 37008 Salamanca, Spain; eddybetances2011@usal.es; 2 Food Technology, University of Salamanca, E.P.S. de Zamora, AvenidaRequejo 33, 49022 Zamora, Spain; irevilla@usal.es (I.R.); avivar@usal.es (A.M.V.-Q.)

**Keywords:** propolis, NIR spectroscopy, flavonoids, antioxidant capacity

## Abstract

The use of propolis as a dietary supplement or as an ingredient in different food products is increasing, due to its antioxidant and bactericidal properties. These nutritional properties directly depend on its phenolic composition. For this reason, this study analysed the total contents of flavones and flavonols, flavanones and dihydroflavonols, and the antioxidant capacity by using the methods of ABTS and linoleic acid/*β*-carotene in 99 samples of propolis from Spain and Chile. A rapid method was developed for quantifying these parameters in raw propolis using near infrared (NIR) spectroscopy with a remote reflectance fibre-optic probe applied directly to the ground-up sample. The models developed allow for the determination of the total flavones and flavonols (0–183 mg quercetin/g propolis and 0–72 mg rutin/g propolis), of the total flavanones and dihydroflavonols (9–109 mg pinocembrin/g propolis extract), and of its antioxidant capacity by the ABTS method based on the reduction of the 2.2-azinobis(3-ethylbenzothiazoline-6-sulfonic acid) radical cation(0–3212.6 nmol Trolox/mg of propolis) and of linoleic acid/*β*-carotene (22–86% inhibition). The NIR spectroscopy models were applied in external validation to different samples of the calibration group, which led to the conclusion that the methods developed provide significantly identical data to the initial chemical data of reference.

## 1. Introduction

Propolis is a resinous substance collected and transformed by honeybees from buds and plant wounds. They use exuded resins in addition to substances actively secreted by plants, including lipophilic materials from leaves and leaf buds, and gums and lattices. Propolisis used in the hive to reinforce its structural integrity, to seal entrances during winter, to reduce vibrations, and as an antiseptic agent. The composition of propolis is very complex and varies depending on the phytogeographic diversity of the area where it is collected and the specific time of year [1,2,3,4]. It is well known that in the detailed chemical composition of propolis the presence of biologically active compounds such as polyphenols, flavonoids, phenolic acids and their esters stands out, which justifies many of its healthy properties for human consumption [5,6]. In recent decades, propolis has attracted a great deal of attention and is being used in foods, beverages, dietary supplements, and cosmetics owing to its antioxidant, antimicrobial, anti-inflammatory, and immunostimulating properties [7,8,9,10].

In the bibliographical record of the methods of quantification of the species of interest to this study, the spectrophotometric determinations of flavones and flavonolsthat use quercetin or rutin as a reference are noteworthy, in accordance with the method proposed by Bonvehí et al. (1994) [11], which has been adopted by other researchers [12,13]. The presence of flavones and dihydroflavones is determined by spectrophotometric methods by means of the reaction with 3.4-dinitrophenylhydrazine (DNP) andpinocembrin as a reference [12,14,15]; however, the use of chromatographic techniques should also be stressed [12,16,17,18]. On the other hand, the determination of the antioxidant capacity in propolis is measured by using the inhibiting activity of the ABTS (2.2-azinobis(3-ethylbenzothiazoline-6-sulfonic acid) [19,20,21,22] and the linoleic acid/*β*-carotene radical [19,23,24,25,26,27], withTrolox as a method of reference. These methods are specific to each compound and time-consuming; and they need important amounts of polluting solvents, while NIR technology is known for being a multiparametric, rapid, and non-destructive technique. It has been used in the characterisation of Mexican propolis with FTIR UV-Vis techniques [28], in the determination of chrysin and galangin in Chinese propolis [29], in the detection of the adulteration of propolis with Poplar balata [30], in the identification of beeswax in this product [31], in the assessment of the mineral composition [32], and in more recent applications such as the estimation ofcaffeic acid phenylethyl ester or CAPE [33] or the determination of pest control substances in propolis [34].

The objective of this study was to develop a quick method to quantify the composition of flavones and flavonols, flavanones, and dihydroflavonols in propolis, and its antioxidant capacity using near infrared spectroscopy (NIR) with a reflectance fibre-optic probe applied directly to the ground up sample of propolis, using samples from Spain and Chile.

## 2. Materials and Methods

### 2.1.Samples

Samples of propolis (99 samples) were directly collected by beekeepers in Chile (the Bio-Bio region, 52 samples) and Spain (Galicia, 14 samples and Castilla y León, 33 samples). The Chilean and Spanish regions included in this study show a great uniformity of both geographical and environmental characteristics. All the regions are consideredtemperate zones according to the Köppen climate classification. However, Bio-Bio and Galicia have temperate summers while Castilla y León has warm summers. *Populus* spp., is the main plant source used by bees in all the regions studied.

The samples were collected mostly with a mesh and with the scraping technique from different beekeepers. Sampleswere ground up in a Foss Knifetec1095 grinder (Höganäs, Sweden), their NIR spectra were recorded, and all samples were kept frozen until used in the laboratory. When using the solutions of the propolis, extracts are prepared according to the method reported by [11,12,13,18,35], with slight modifications. Ten milliliters of methanol were added to a 1 g aliquot of sample, and extraction was subsequently carried out in an ultrasonic bath for 15 min. The methanol extract was centrifuged (1500 rpm) for 10 min at 20 °C. The supernatant was filtered through Whatman grade 4 filter paper and the resulting liquid was transferred to a 10 mL volumetric flask. This methanolic extract was diluted 1:100 with methanol for its analytical determination.

### 2.2. Chemical Methods

#### 2.2.1. Flavones and Flavonols

The content of flavones and flavonols is quantified as described by the authors of [11,12,13,18,35], with minor modifications. A solution of AlCl_3_ in ethanol (0.5 mL) is added to 2 mL of propolis alcoholic extract. After 30 min at room temperature, the absorbance at 425 nm is measured. The results were expressed in milligrams of quercetin or rutin (used as a reference) per gram of propolis, using the calibration lines drawn up with each of said standards for this purpose.

#### 2.2.2. Flavanones and Dihydroflavonols

The total quantification of flavanones and dihydroflavonols is carried out according to the method described by Popova et al. (2004) [12], with minor modifications. A 1 mL aliquot of sample of propolis alcoholic extract and 2 mL of the DNP solution (2.4-dinitrophenylhydrazine) (solution: 1 g DNP in 2 mL of 96% sulphuric acid, diluted to 100 mL with methanol) is heated at 50 °C for 50 min. After it has cooled to room temperature, 10% potassium hydroxide (KOH) in methanol (w/v) to 10 mL is added to the solution. A total of 1 mL of the resulting solution is diluted to 50 mL with methanol in a volumetric flask and the absorbance is measured at 486 nm. The results are expressed as milligrams of pinocembrin (used as a reference) per grams of propolis extract using the corresponding calibration curve.

#### 2.2.3. Antioxidant Activity, Inhibiting Capacity of the ABTS Radical

The total antioxidant capacity was determined with the ABTS method, which is based on the reduction of the 2.2-azinobis(3-ethylbenzothiazoline-6-sulfonic acid) radical cation. Scavenging of the ABTS+ radical was monitored by the decrease in absorbance at 734 nm by spectrophotometry [36]. The water-soluble vitamin E analogue Trolox (6-hydroxy-2,5,7,8-tetramethylchorman-2-carboxylic acid) was used as standard. To prepare the ABTS radical cation, an ABTS solution was oxidized in water by treating it with potassium persulfate (molar ratio = 1:0.35) for 12–16 h in the dark, and then diluted in a 2 mL cuvette with 0.1 M potassium phosphate buffer, pH 7.4, prior to the assays, to give an absorbance of 0.7 + 0.02 at 734 nm. A suitable amount of the sample (20 μL) was added to the reagent and the mixture was incubated at 25 °C. Absorbance was recorded each minute for 10 min using a Shimadzu spectrophotometer (Columbia, MD, USA). Appropriate solvent blanks were run in each assay. The percentage of inhibition of absorbance at 734 nm was calculated and plotted as a function of the concentration of Trolox to give the Trolox equivalent antioxidant capacity (TEAC).

#### 2.2.4. Inhibiting Activity of the Linoleic Acid/*β*-Carotene Radical

In order to assess the antioxidant capacity of propolis by using the linoleic acid/*β*-carotene method, it is necessary to proceed according to the method described by Emmons et al. (1999) [37], with some modifications. The *β*-carotene (3 mg) is dissolved in 30 mL of chloroform and 3 mL are added to 40 mg of linoleic acid and 400 mg of Tween 40. The chloroform is eliminated under a rotary evaporator and 100 mL of distilled water are added; and the solution is mixed well. The aliquots (3 mL) of the linoleic acid/*β*-carotene emulsion are mixed with 50 µL of propolis ethanol extract and are incubated in a thermostatic water bath at 50 °C. The oxidation of the emulsion is monitoredspectrophotometrically by measuring the absorbance at 470 nm during a 60 min period. The results are expressed as the inhibition percentage of the spectrophotometric signal which is calculated by using the following formula: [(A_0_ − A_1_/A_0_) × 100], in which (A_0_) is absorbance at time zero and (A_1_) is absorbance after 60 min.

### 2.3. NIR Spectroscopy

A Foss NIRSystem 5000(DK-3400, Hillerød, Denmark), with a standard 1.5 m, 210/210 bundle fiber-optic probe, Ref No R6539-A, was used. The spectral range was set at 1100–2000 nm since above this value (2000 nm) significant attenuation of the signal occurred due to strong absorption of the OH groups present in the optical fiber. The probe employed a remote reflectance system and used a ceramic plate as a reference. The window was made of quartz with a 5 cm × 5 cm surface area. The NIR spectrum was obtained for each of the samples by applying the remote reflectance fiber-optic probe to ground-up propolis. The spectra were recorded at 2 nm intervals, and 32 scans were taken for both the reference and the samples. All samples were analyzed in triplicate in order to minimize sampling errors. For subsequent statistical analysis, 70 propolis samples were randomly selected for the calibration set, while the remaining 29 samples formed the validation set.

### 2.4. Chemometric Methods, NIR-Chemometric Methods

The models of calibration were developed by using the data obtained from analytical determinations and the spectral data obtained from NIR spectra of 99 samples were assessed, of which 70 constitute the calibration group and 29 the external validation set. The samples were selected at random. The quantification of the different analytical parameters was performed using the modified partial least squares (MPLS) regression method. Partial least squares (PLS) regression is similar to principal component regression (PCR), but uses both reference data (chemical, physical, etc.) and spectral information to form the factors that may be useful for fitting purposes [38]. MPLS is often more stable and accurate than the standard PLS algorithm. In MPLS, the NIR residuals, obtained after each factor and at each wavelength, were calculated and standardized (dividing by the standard deviations of the residuals at each wavelength) before the next factor was calculated. The scattering effects were removed using multiplicative scatter correction (MSC), standard normal variate (SNV), DeTrend (DT) or SNV–DT. Moreover, the mathematical treatments were tested in the development of the NIRS calibrations by using a nomenclature of 2,4,4,1 in which the first digit is the number of the derivative, the second is the gap over which the derivative is calculated, the third is the number of data points in a running average or smoothing, and the fourth is the second smoothing. When developing the MPLS equations, cross-validation is recommended in order to select the optimal number of factors and to avoid overfitting [39,40], and the calibration set is divided into several groups for the cross-validation. Each group is then validated using a calibration based on the other samples. Finally, any validation errors generated are combined into a root mean square error of cross-validation (RMSECV) [41].

## 3. Results and Discussion

### 3.1. Chemical Analyses and Spectral Information

**Figure 1:**
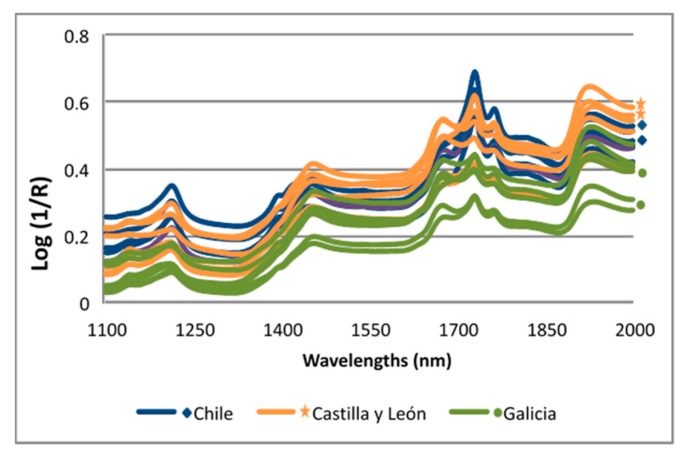
NIR spectra of propolis from Chile and Spain (Castilla y León and Galicia).

When the spectra of the propolis samples from different geographical areas (Galicia, Castilla y León, Chile) are observed, their variability can be appreciated according to these different origins (Figure 1). This spectral variability is suitable for the development of NIR calibration models of the parameters under study. The differentiation of the spectra of the propolis according to its origin has been revealed in the study of González-Martín et al. [34].

The composition of the 99 samples of propolisanalyzedis shown in Table 1, which includes the contents of flavones and flavonols, flavanones and dihydroflavonols, the inhibiting activity of the ABTS radical and the antioxidant activity on linoleic acid oxidation. It shows the minimum and maximum values, the mean, and the standard deviation (SD) for each of the constituents by regions and countries. In the quantification of flavones and flavonols, quercetin and rutin have been used as a reference owing to the fact that various authors use both compounds for their determination; in this study, both have been used to compare the results obtained with the bibliographical sources consulted.

The content in flavones and flavonols, regardless of whether quercetin or rutin is used as a reference, is found in larger amounts in Castilla y León (8.0–49.9 mg quercetin/g of propolis), (19.9–190.3 mg rutin/g of propolis), followed by Galicia (10.4–58.7 mg quercetin/g of propolis), (28.2–149.6 mg rutin/g of propolis) and Chile (0–63.3 mg quercetin/g of propolis), (0–161.1 mg rutin/g of propolis). In the case of the content of flavanones and dihydroflavonols referring to pinocembrin, the highest content is to be found in Castilla y León (31.7–81.6 mg pinocembrin/g of propolis), followed by Galicia (31.8–73.9 mg pinocembrin/g of propolis) and Chile (27.0–149.7 mg pinocembrin/g of propolis). However, in the case of the antioxidant activity determined by the ABTS method the highest value is to be found in the Chilean samples with 641.2–8215.4 nmolTrolox/mg propolis, followed by Castilla and León (1197.7–2649.3 nmolTrolox/mg propolis) and Galicia (1552.1–2012.6 nmolTrolox/mg propolis). In the same manner, the inhibiting capacity of linoleic acid/*β*-carotene is greater in Chile (54.7–88.1% inhibition), in this case followed by Galicia (72.7–83.3% inhibition), and the lowest value occurs in Castilla y León (21.6–82.2% inhibition). The values found for flavones and flavonols were in general higher than those found in Chinese samples [19], by authors who used quercetin as a reference, while the values of this study are lower than those found in propolis from Mexico when using rutin as a reference [13].

With regard to the contents of flavanones and dihydroflavonols, results found in Spanish propolis samples are higher than those found in propolis from Mexico [13], Portugal [15], and Argentina [25]. As far as antioxidant activity is concerned, the closest ABTS values to those found in this study were found in Turkish propolis [20]. Finally, the results of the antioxidant capacity determined by the linoleic acid/*β*-carotene method of this study are in line with those found in various locations in China [19], and similar to those found by Kumazawain countries such as Argentina [23], Australia, Chile, China, and Hungary, which are higher than propolis values from Brazil [26] and Korea [27].

### 3.2. NIR Calibration Equations

The NIR data are divided into two established groups: 70 samples serve to constitute the calibration set and 29samples are used for the external validation set, chosen always at random.

Prior to the application of the Modified Partial Least Squares (MPLS) regression model, the samples with a value of H (the Mahalanobis distance) greater than 3 are eliminated. Subsequently the MPLS regression is carried out; those samples with T values exceeding 2.5 are eliminated from the set because they are different from the population from a chemical point of view. The results of the NIR calibration models obtained for each constituent can be seen in Table 2 with the indication of the number of samples (N) used (after eliminating the samples because of criterion H and criterion T), together with the best of the different mathematical treatments, the range of concentration, standard deviations for each parameter, R^2^ values, and calibration errors (SEC). The equations obtained allow the determination of the flavones and flavonols (taking both quercetin and rutin as a reference), flavanones and dihydroflavonols, the inhibiting activity of the ABTS radical, and the antioxidant activity on linoleic acid oxidation.

N = number of samples analyzed. SEC = Standard calibration error. SECV = cross-validation standard error. SD = standard deviation. R^2^ = determination coefficient. RMSEP = mean square error of prediction. C_NIR_ = NIR concentration. C_Ref_ = Reference concentration. Samples deleted by criterion H and criterion T (respectively): Total flavones + flavonols (mg quercetin/g propolis) (4 and 1); Total flavones + flavonols (mg rutin/g propolis) (5 and 0); Total flavanones + dihydroflavonols (mg pinocembrin/g propolis) extract (6 and 2); ABTS (nmolTrolox/mg propolis) (5 and 2); Linoleic acid/*β*-carotene (% inhibition) (3 and 1). Latent variables: 7 (Total flavones + flavonols (mg rutin/g propolis), Total flavanones + dihydroflavonols (mg pinocembrin/g propolis extract) and ABTS; 8 Total flavones + flavonols (mg quercetin/g propolis) and Linoleic acid/*β*-carotene (% inhibition).

The results obtained indicate that given the high R^2^ values and the small calibration errors it is possible to determine the flavone and flavonol contentby means of NIR technology independently of the standard compound used for calibration, and the total flavanone and dihydroflavonol in concentrations similar to those found in spectrophotometry. For the determination of antioxidant activity using the ABTS method, the concentration margin is lower (0–3212.6 nmol of Trolox/mg of propolis), while in the case of thelinoleic/*β*-carotene method the margin of application of the model regarding the chemical data of reference clearly widens (22.7–86.8% inhibition).

### 3.3. Internal Validation (Prediction)

**Figure 2:**
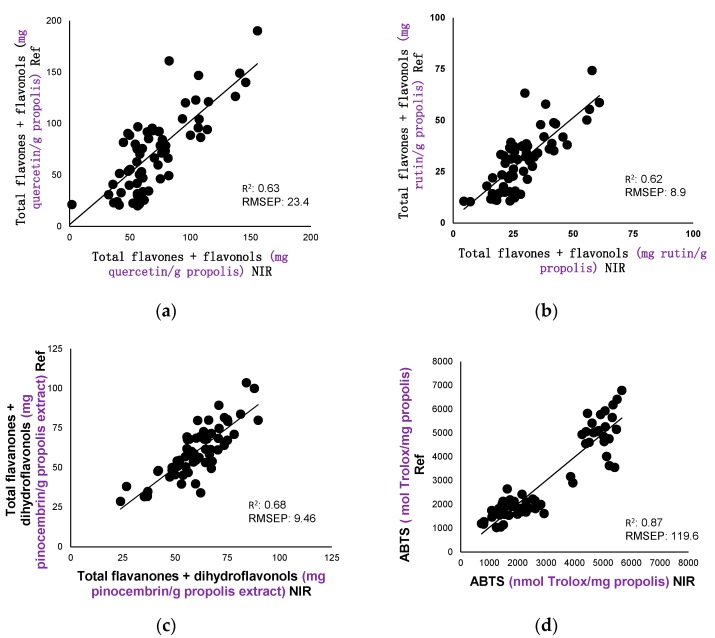

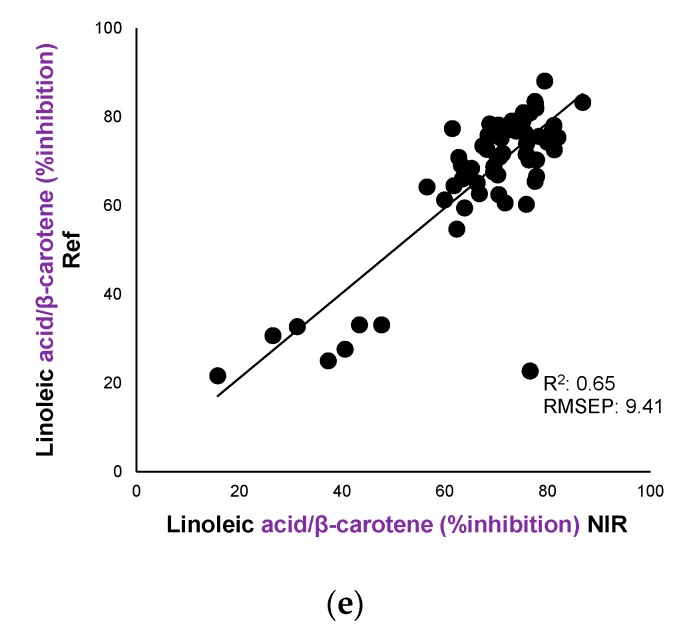
Comparison of the reference values with the values predicted by calibration equations NIR. R^2^ = determination coefficient; RMSEP = mean square error of prediction.

The models obtained by NIR calibration are assessed by cross-validation. The set of calibration samples was divided into a series of subsets (establishing seven cross-validation groups). The prediction process involves taking six of these sets for the calibration set and one for the prediction set. The process is repeated for each subset so that all the samples pass the calibration set and the prediction set. It can be seen from Table 2 that the cross-validation errors (SECV) are of the same kind as those of calibration. This table indicates the regression lines of NIR calibration compared with the reference data obtained by spectrophotometry. This method allows the validation of the models obtained as well as the checking of their prediction capacities. The correlations of the values obtained in the laboratory (Ref) with regard to those predicted by NIR with a fibre-optic probe of the flavones and flavonols (referring to mg of quercetin or of rutin/g of propolis), flavanones and dihydroflavonols (referring to mg pinocembrin/g of propolis), the inhibiting activity of the ABTS radical (nmolTrolox/mg propolis) and the antioxidant activity on the linoleic acid (% inhibition) are shown in Figure 2. These data tell us that the NIR models obtained can be used to predict these parameters in unknown samples. NIR technology with a fibre-optic probe may become an alternative to the chemical methods used. This spectroscopic method has great potential owing to its low cost, as it does not require the treatment of the samples compared with chemical methods.

### 3.4. External Validation

Once the NIR calibration equations have been obtained for the determination of the composition of the total of flavones and flavonols, flavanones and dihydroflavonols, the inhibiting activity of the ABTS radical and the antioxidant activity on linoleic acid oxidation in propolis, it is necessary to proceed to the stage of external validation, which consists of the application of the equations to a set of 29 samples that do not belong to the calibration set. The procedure is as follows: the NIR spectra are recorded in triplicate and the spectral mean is taken.

The NIR calibration equations obtained in the study are applied to said spectra to predict the values for each of the parameters; subsequently they are compared with the results predicted by means of NIR technology with the laboratory chemical data of these samples.Student’s *t*-testis used to compare both methods (spectrophotometry and NIR). Table 3 shows the results obtained in the external validation, the residual means, the Root Mean Square Error (RMSE), and R^2^. The NIRS and chemical methodologies were compared for all constituents using Student’s *t*-test for paired values with these samples. The levels of significance were found to be 0.41 for ABTS, 0.59 for the total of flavanones and dihydroflavonols, 0.97 for flavones and flavonols, and 1.00 for the linoleic acid/*β*-carotene method. The level of significance for all constituents was higher than 0.05 (chosen as the minimum), i.e., there were no differences between the results obtained. It may therefore be concluded that the method significantly provides equal data to the starting reference data.

The RMSE values were lower than the standard calibration error (SEC) for all the compounds studied: Total flavones + flavonols (mg quercetin/g propolis) (6.6 < 24.1); Total flavones + flavonols (mg rutin/g propolis) (3.9 < 9.5); Total flavanones + dihydroflavonols (mg pinocembrin/g propolis extract) (4.1 < 10.2); ABTS (−68.5 < 386.1) and linoleic acid/*β*-carotene (3.8 < 72.3). For antioxidant activity determined by linoleic acid/*β*-carotene method the prediction could have limitations in the lower part of the interval due to the irregular distribution of reference data (Table 1). This is the reason why the SEC value was high (72.3) although the RMSEP was low (9.41). Regarding the residual mean values, they were suitable for this kind of methodology. The R^2^ values for external validation have the same magnitude as those obtained in the calibration; it is pointed out that when the models developed for NIR technology are applied to unknown samples the results are satisfactory.

It can be emphasised that up to now we have found no research implementing NIR technology in propolis for the determination of these parameters.

The regression model used can be justified by means of the correlation between the concentration and the different wavelengths given by the values of the *β* coefficients that are obtained from calculating the parameters of the equation:

(1) $$y=\beta_{0}+\beta_{1}X_{\lambda_{1}}+\beta_{2}X_{\lambda_{2}}+\beta_{3}X_{\lambda_{3}}+...\beta_{n}X_{\lambda_{n}}$$

in which *β*_0_, *β*_1_, *β*_2_ … are the coefficients and $X_{\lambda_{1}}$, $X_{\lambda_{2}}$, $X_{\lambda_{3}}$... are the wavelengths in which the correlation with the concentration of the variables (parameters studied) shows the maximum reflectance. The data for the component at the most significant wavelengths can be seen in the new Table 4.

## 4. Conclusions

In view of the results, NIR methodology can be used to predict the total contents offlavonesand flavonols, the sum offlavanones anddihydroflavonols, and the inhibiting activity of the ABTSradical and the antioxidant activity on linoleic acid oxidation in propolis with values comparable to spectrophotometry. The most determinant aspect of this methodology is that it can be developed and applied to any type of unknown propolis of different origins without prior treatment and without destruction of the samples, i.e., from the direct application of the fibre-optic probe after grinding up the propolis.

## Figures and Tables

**Table 1:** Chemical data on flavones and flavonols, flavanones, and dihydroflavonols, the inhibiting activity of the ABTS (2.2-azinobis(3-ethylbenzothiazoline-6-sulfonic acid) radical and the antioxidant activity on linoleic acid oxidation.

Chemical Data Obtained by Spectrophotometry									
Countries	Spain						Chile		
Regions/n° of Samples	Galicia/14			Castilla y León/33			Bio-Bio/52		
Constituents	Min–Max	Mean	SD	Min–Max	Mean	SD	Min–Max	Mean	SD
Total flavones + flavonols (mg quercetin/g propolis)	28.2–149.6	61.5	36.3	19.9–190.3	88.5	38.9	0–161.1	57.3	35.6
Total flavones + flavonols (mg rutin/g propolis)	10.4–58.7	25.8	13.7	10.1–74.3	35.9	14.6	0–63.3	24.1	13.6
Total flavanones + dihydroflavonols (mg pinocembrin/g propolisextract)	31.8–73.9	49.4	9.6	31.7–81.6	56.7	15.3	27.0–149.7	75.8	34.3
ABTS (nmolTrolox/mg propolis)	1552.1–2012.6	1777.1	195.1	1197.7–2649.3	1907.7	384.2	641.2–8215.4	3863.5	1911.2
Linoleic acid/*β*-carotene (% inhibition)	72.7–83.3	78.0	3.7	21.6–82.2	56.5	21.1	54.7–88.1	70.0	7.1

**Table 2:** NIR calibration data of the 70 samples of each of the flavones and flavonols, the flavanones and dihydroflavonol, and the inhibiting activity of the ABTS radical and the antioxidant activity on linoleic acid oxidation.

Constituents	Mathematical Treatment	N	Min–Max	SEC	SECV	SD	R^2^	RMSEP	Regression Line
Total flavones + flavonols (mg quercetin/g propolis)	Standard MSC 2,4,4,1	65	0–183.4	24.1	29.4	37.9	0.63	23.4	C_NIR_ = 1.00 C_Ref_ + 1.78
Total flavones + flavonols (mg rutin/g propolis)	Detrend only 0,0,1,1	65	0–72.0	9.5	11.8	14.4	0.62	8.9	C_NIR_ = 0.98 C_Ref_ + 2.33
Total flavanones + dihydroflavonols (mg pinocembrin/g propolis extract)	Standard MSC 2,4,4,1	62	9.89–109.4	10.2	13.4	16.6	0.68	9.5	C_NIR_ = 1 C_Ref_ + 0.00
ABTS (nmolTrolox/mg propolis)	Detrend only 2,10,10,1	63	0–3212.7	386. 1	449.3	707.7	0.87	119.6	C_NIR_ = 0.99 C_Ref_ + 44.03
Linoleic acid/*β*-carotene (% inhibition)	SNV only 1,4,4,1	66	22.7–86.8	72.3	139	15.0	0.65	9.41	C_NIR_ = 0.96 C_Ref_ + 1.9

N = number of samples analyzed. SEC = Standard calibration error. SECV = cross-validation standard error. SD = standard deviation. R^2^ = determination coefficient. RMSEP = mean square error of prediction. C_NIR_ = NIR concentration. C_Ref_ = Reference concentration. Samples deleted by criterion H and criterion T (respectively): Total flavones + flavonols (mg quercetin/g propolis) (4 and 1); Total flavones + flavonols (mg rutin/g propolis) (5 and 0); Total flavanones + dihydroflavonols (mg pinocembrin/g propolis) extract (6 and 2); ABTS (nmolTrolox/mg propolis) (5 and 2); Linoleic acid/*β*-carotene (% inhibition) (3 and 1). Latent variables: 7 (Total flavones + flavonols (mg rutin/g propolis), Total flavanones + dihydroflavonols (mg pinocembrin/g propolis extract) and ABTS; 8 Total flavones + flavonols (mg quercetin/g propolis) and Linoleic acid/*β*-carotene (% inhibition).

**Table 3:** Data of external validation (29 samples), level of significance, residual means, root mean square error (RMSE), and R^2^ of flavones and flavonols, flavanones and dihydroflavonols, and inhibiting activity of the ABTS radical and antioxidant activity on linoleic acid oxidation in propolis.

Constituents	*p* (Level of Significance)	Residual Mean	RMSEP	R^2^
Total (flavones + flavonols) (mg quercetin/g propolis)	0.97	98.8	6.6	0.60
Total (flavones + flavonols) (mg rutin/g propolis)	0.97	74.1	3.9	0.61
Total (flavanones + dihydroflavonols) (mg pinocembrin/g propolis extract)	0.59	26.7	4.1	0.63
ABTS (nmolTrolox/mg propolis)	0.41	45.2	68.5	0.86
Linoleic acid/*β*-carotene (% inhibition)	1.00	20.4	3.8	0.64

**Table 4:** Correlation between the concentration of each constituent at the most significant wavelengths.

Total (Flavones + Flavonols) (mg Quercetin/g Propolis		Total (Flavones + Flavonols) (mg Rutin/g Propolis)		Total (Flavanones + Dihydroflavonols) (mg Pinocembrin/g Propolis Extract)		ABTS (nmolTrolox/mg Propolis)		Linoleic acid/*β*-Carotene (% Inhibition)	
*λ* (nm)	*β*	*λ* (nm)	*β*	*λ* (nm)	*β*	*λ* (nm)	*β*	*λ* (nm)	*β*
1282	956.2	1266	354.2	1156	63.6	1218	7754.0	1542	110,178.8
1304	919.5	1752	186.6	1454	65.8	1454	4155.6	1828	115,808.4
1750	835.6	1540	−571.3	1594	−210.2	1408	−8605.2	1962	148,099.2
1540	−2899.0	1848	−516.7	1796	−97.6	1796	−8331.7	1512	−158,593.2
1856	−2290.6							1812	142,605.7
